# Neuropsychological monitoring of cognitive function and ICF–based mental components in patients with malignant brain tumours

**DOI:** 10.3389/fpsyg.2023.1033185

**Published:** 2023-03-31

**Authors:** Agnieszka Pilarska, Anna Pieczyńska, Katarzyna Hojan

**Affiliations:** ^1^Department of Rehabilitation, Greater Poland Cancer Centre, Poznan, Poland; ^2^Department of Occupational Therapy, Poznan University of Medical Sciences, Poznan, Poland

**Keywords:** brain cancer, cognitive rehabilitation, cognitive assessment tools, neuropsychological assessment, international classification of functioning, ICF

## Abstract

**Background:**

Cognitive deficits are one of the important clinical features of patients with brain tumours, which can affect up to 30–90% of patients before treatment. The consequence is a significant and rapid degradation of the patient’s intellectual functioning, seizures, paralysis and other symptoms that prevent independent functioning. This results in a reduced quality of life and a psychological crisis not only for the patient but also for their relatives. Maintaining the patient’s function at the highest level for as long as possible is particularly important, given that long-term remission or a cure is unlikely or accompanied by significant disability.

**Purpose:**

This paper aims to provide a narrative review to the neuropsychological procedure for monitoring cognitive function in patients with brain tumours, which may be helpful in developing adequate clinical practice and appropriate management procedures.

**Methods:**

A narrative review was applied to search broadly across disciplines, retrieving literature from several databases (PubMed, Web of Science, and EBSCOhost).

**Results:**

(1) discussing the methodological aspects of neuropsychological tools for monitoring cognitive function in brain tumour patients, (2) identifying the most commonly used tools and (3) their practical applicability according to the cognitive function components of the International Classification of Functioning, Disability and Health (ICF).

**Conclusion:**

This article points to the need to systematise research tools or develop new ones, adapted to diagnostic needs with high psychometric characteristics, with particular attention to memory processes and learning effect. Rehabilitation of patients is also an important issue, which requires the use of adequate tools to assess functional disability. The International Classification of Functioning, Disability and Health (ICF) seems to be useful in this respect. The ICF has the advantage of targeting actions to improve the condition of the individual and to keep them as long as possible in a state of well-being that allows them to function effectively in society or to return to work. This is particularly important in view of the ageing population and the increasing number of diagnoses related to brain tumours.

## 1. Introduction

Cognitive deficits are one of the important clinical features of patients with brain tumours which can affect up to 30–90% of patients before treatment (Taphoorn and Klein, 2004). The degree of impairment is correlated with the volume of tumour tissue, number of metastatic lesions and their location (Zucchella et al., 2013; Pendergrass et al., 2018). It may be also related to the variability of the tumour itself, the use of different neuropsychological tools for testing, variable cut-off points and normative data. Cognitive deficits may relate to attention, memory and executive functioning (Van Coevorden-van Loon et al., 2015) and may be a marker of tumour progression before clinical or imaging symptoms appear (Tallet et al., 2012; Durand et al., 2015).

Depending on the area in which the tumor is located, the patient presents certain, specific deficits in cognitive and other areas (Gould, 2018). A tumor located in the frontal lobe can impair voluntary movements, speech fluency, emotional control, as well as executive functions and memory. The parietal lobe, on the other hand, has different roles in the left and right hemispheres of the brain. Left lobe tumors often impair speech, including the understanding of symbolic language, and right lobe tumors may affect the perception of the physical location of body parts and the understanding of geographical location (Gould, 2018; D’Souza et al., 2021). Tumors located in the temporal lobes can cause disorders not only in the processing of auditory sensations, but also in speech, verbal memory, recognition of objects and faces, and smells (Yamamoto et al., 2022). Tumors in the occipital lobe can cause blindness, blurred vision, and hallucinations (Hense et al., 2021). Cerebellar tumors affect the ability to coordinate voluntary movements such as balance and blinking whereas brain stem tumors are responsible for disorders of many functions, including basic ones, such as paresis of the limbs, speaking, swallowing, breathing or seeing (Reyes-Botero et al., 2012; Beuriat et al., 2022).

Generally, the consequence of the brain tumours is a significant and rapid degradation of the patient’s intellectual functioning, seizures, paralysis and other symptoms that prevent independent functioning. This results in a reduced quality of life and a psychological crisis not only for the patient, but also for their relatives. In this context, maintaining the patient’s function at the highest level for as long as possible is particularly important, given that long-term remission or a cure is unlikely or accompanied by significant disability (Meyers and Brown, 2006).

The report of the *National Cancer Institute (NCI) Brain Tumor Progress Review Group* recommends that the routine assessment of cognitive ability and quality of life (QOL) should become the standard of care for patients with brain tumours. Unfortunately, monitoring presents many difficulties in clinical practice, and one of the key issues is the lack of a consistent methodological framework for conducting the study. Neuropsychological assessment is challenging as it requires knowledge of several disciplines, including knowledge of clinical interviewing, neuroanatomy and neuropsychological symptoms. Knowledge of test administration and interpretation and psychometrics is also required. The selected measures of cognitive functioning should be psychometrically sound, with established reliability as well as validity and appropriate normative tests (Correa, 2006).

A number of psychological tools are commonly used for psychological diagnosis in the course of brain cancer. They measure a variety of cognitive functions, including attention, concentration, memory, language and executive skills. Most of them are similar in terms of the time and type of task performance, engagement of specific cognitive functions and psychometric properties. So far, there is a lack of specific test batteries, universal for any medical centre. In addition, there are similar difficulties in assessing the needs and outcomes of rehabilitation patients, which require an adequate tool to assess functional disability (Ģiga et al., 2021). There are not many standardised assessment protocols for people with various brain tumours. A basis for the comparison of functional assessment tools could be the International Classification of Functioning, Disability and Health (ICF), which provides a common standard language for identification and offers a framework for the large-scale coding of health information. By combining information on clinical diagnosis (according to the ICD-11 Classification) and functioning (ICF), a broader and more meaningful picture of the health of individuals and populations can be achieved for better decision-making. This approach was proposed by Khan and Amatya (2013) who described patient-reported disability in primary brain tumours using ICF and compared with categories within the core sets for stroke and traumatic brain injury. Their findings would assist in defining a future core set for brain tumour, however, using a single core set relevant to most long-term neurological conditions needs to be explored. Recently, ICF classification system has been proposed to describe how professionals in healthcare, habilitation, and school might document problems with everyday life functioning at body, activity, and participation levels for children who completed treatment for a brain tumor (Björklund et al., 2021). Additionally, method to establish comparability of health information based on the ICF was developed by other group. This method based on Linking Rules involve preparing information for linking, perspectives from which information is collected and the categorization of response options (Cieza et al., 2019).

The present paper aims to provide a narrative review to the neuropsychological procedure for monitoring cognitive function in patients with brain tumours, which may be helpful in developing adequate clinical practice and appropriate management procedures by (1) discussing the methodological aspects of neuropsychological tools for monitoring cognitive function in brain tumour patients, (2) identifying the most commonly used tools and (3) their practical applicability according to the cognitive function components of the ICF.

## 2. Methodological notes on cognitive testing in patients with brain tumours

### 2.1. General comments

Assessment of cognitive function is an important and necessary part of the contemporary, comprehensive oncology care for cancer patients especially with brain tumours. Cancer-related cognitive impairment may result from the direct effects of the cancer itself, non-specific factors or comorbidities that are independent of the disease or result from adverse effects of treatment or treatment combinations used in conjunction with the disease. The prevalence and extent of cognitive impairment associated with cancer are known but not well understood, in part due to significant differences in the research methods and definitions used to assess cognitive functioning in these patients (Pendergrass et al., 2018).

A properly applied research method should be based on the possibility that there is a reliable relationship between observable behavioural changes and the presence of a focal region of brain damage with a specific anatomical position. Procedural errors, such as the use of inappropriate neuropathological samples or inappropriate composition of study groups, can severely limit the usefulness of the method (Damasio et al., 1990).

Despite a large increase in the number of clinical trials, cognitive decline is difficult to study in patients—not only for methodological reasons but also because of the relatively small sample sizes, differences in the patients’ age, the nature and location of the tumour(s), additional anticancer treatment (e.g., hormonal) and the intensity of adjuvant treatment (Seigers and Fardell, 2011). There is also wide variation across the used method in clinical studies in the neuropsychological tests used and the criteria used to determine cognitive impairment. Furthermore, not every study uses appropriate control groups, study designs or statistical measures (Schagen et al., 2014).

In most clinical trials involving adult patients with brain tumours, assessment of cognitive function is based on performance status, mental status examination, patient complaints and clinical observation (Corry et al., 1998). These methods are known to have low sensitivity in detecting cognitive impairment in patients with brain tumours (Correa et al., 2007). These studies can only show significant cognitive decline, suggesting that the prevalence of cognitive impairment in patients with brain tumours is underestimated (Correa, 2006).

Therefore, it is important to use neuropsychological tests that are sensitive to mild changes in cognitive function, have multiple versions or are robust to learning effects. Mood and QOL scales and assessment of estimated pre-disease intelligence quotient (IQ) should also be used here. In turn, given the need to minimise the effects of fatigue, sessions should be short and not exceed 1  hour. In addition, to guarantee data quality and consistency between medical centres, training and certification of professionals involved in test administration and supervision by a neuropsychologist are essential (Correa, 2006).

Neuropsychological supervision should concern not only the knowledge of reliability, validation and appropriate use of tests but, above all, the ability to interpret their results. Interpretation of the test results is a complex process, as many factors—both on the patient’s side and contextually—can influence the result obtained. For example, in a memory function test situation, it is not only the patient’s attention, language or executive functioning that influences the result, but also the level of stress or fatigue experienced.

Understanding the above issues regarding the test and patient factors that may undermine the reliability of test results plays a key role in the reliability and safety of the testing procedures performed (Meyers and Brown, 2006).

### 2.2. Analysis of the treatment process

The traditional outcome measures used in brain tumour research include the overall and recurrence-free survival of patients and, in some cases, respond to treatment with chemotherapy and radiotherapy. Such an analysis of the treatment outcomes usually neglects the aspects related to patients’ quality of life and the biological response to treatment. From a practical point of view, these issues are important in two situations: (1) for brain tumours, where there are currently no effective treatment therapies; (2) for tumours detected in children, where tumour control is linked to the possibility of the disease being considered chronic. Improved measurement tools would allow for the rapid rejection of potentially neurotoxic therapies and continuation of effective therapies (Brain Tumor Progress Review Group, National Cancer Institute (U.S.) and National Institute of Neurological Disorders and Stroke (U.S.), 2000).

In the context of research questions about the prospective neurocognitive testing of patients, it is, therefore, important to consider: (1) pre-treatment cognitive problems to establish a baseline; (2) data showing which treatment regimens improve neurocognitive function, slow down the expected deterioration or have neurotoxic effects in the shorter and longer term. Neurocognitive diagnostics are also important for diagnostic differentiation (e.g., distinguishing depression from frontal lobe dysfunction) in order to optimise treatment and therapeutic intervention (Meyers and Brown, 2006). Regarding the different treatment modalities and their impact on cognitive function, the literature most often points to surgery, chemotherapy, radiotherapy and, in addition, hormone therapy or immunotherapy.

Studies show that, for the diagnosis of primary brain tumours, extensive surgical resection has a survival benefit (Brown et al., 2016), and after surgery patients also experience fewer seizures, intracranial pressure symptoms and headaches. According to Coomans et al. (2019), the main challenge in patients with brain tumours is to maximise a well-tolerated resection while avoiding severe neurological deficits that impair cognitive functioning.

The treatment of brain tumours with chemotherapy is of limited use, which is related to low primary chemosensitivity, early secondary chemo-resistance, the presence of the blood–brain barrier and the neurotoxicity of some cytostatic drugs and their adverse interactions with other drugs (e.g., anticonvulsants). It is estimated that, among patients receiving chemotherapy, between 13 and 70% exhibit measurable cognitive impairment), some of which may be present even before treatment (Ahles et al., 2010; Wefel et al., 2010, 2011a). Additional challenges in assessing the impact of chemotherapy on cognitive function are the side effects of the concurrent administration of different cytotoxic drugs, which may have a more negative impact on cognitive functioning, and the different reactivity of patients to the effects of the same chemotherapy regimen (Wefel et al., 2011a).

The pathophysiological mechanisms of chemotherapy-induced CNS damage are not well understood but are thought to include microvascular damage, demyelination and a secondary inflammatory response. Acute neurotoxicity may occur during or shortly after treatment with chemotherapy (Keime-Guibert et al., 1998) and fatigue may contribute to mental status changes (Correa, 2006). It is estimated that cancer-related fatigue may affect 90% of patients undergoing cancer treatment. Chronic fatigue as a side effect of chemotherapy may also persist in patients who have recovered from cancer, resulting in long-term cognitive decline, e.g., in attention and working memory (Pendergrass et al., 2018). The causes of this are thought to be an imbalance between the inflammatory and inhibitory mechanisms or changes in brain function. The diagnosis is made on the basis of symptoms of significant, intractable and chronic fatigue, decreased energy or increased need for rest, decreased concentration, decreased motivation to act, insomnia and lack of feeling of relaxation after rest.

Most of the possible complications after chemotherapy pass quickly and do not damage the body. Only a small proportion may be life-threatening (e.g., neutropenic fever) or permanent (e.g., post-anthracycline cardiomyopathy). Such situations require the application of appropriate treatment as well as adequate preventive measures (Keime-Guibert et al., 1998).

An alternative or complementary treatment for patients with brain tumours is radiotherapy. It is often used postoperatively as adjuvant therapy to reduce the risk of local failure, delay tumour progression, prolong survival (in more malignant forms, e.g., glioma) and sometimes as preoperative treatment to avoid serious neurological consequences of surgery (Stieber and Mehta, 2007). Ionising radiation is also often used in the palliative treatment of brain tumours and brain metastases. In the dose range used for the treatment of CNS tumours, the probability of inducing severe late radiation complications and induced secondary tumours is relatively low.

Patients undergoing RT to the brain often experience radiation-induced fatigue and headache as well as possible cognitive impairment. It has been shown that whole brain radiotherapy (WBRT) can exacerbate fatigue (Pendergrass et al., 2018).

Irradiation can lead to significant but mostly transient cognitive impairment (50–90% of patients), occurring in the acute (during irradiation), subacute: early-delayed (in the first months after irradiation) and late (many years after irradiation) phases. Acute and early adverse effects are thought to be transient, whereas late damage may be a risk factor as the associated cognitive impairment may be irreversible and progressive (Coomans et al., 2019).

Some anatomical and functional structures, such as the white matter, hippocampus, cerebellum or temporal and frontal lobes, appear to be sensitive to radiation in human and animal models. The deterioration of their integrity during RT is partly correlated with induced cognitive impairment (Durand et al., 2015).

The subacute toxicity effect is associated with the impairment of information processing, attention, verbal memory, executive functioning and motor skills. Alterations in the white matter are responsible for this, and its recovery after RT may lead to improvements in these functions over time.

The risk factors for the development of cognitive impairment due to irradiation include: an age less than 5 years or greater than 60 years, dose greater than 2 Gy per fraction, higher total dose, hyperfractionated regimens, shorter total treatment time, presence of concurrent vascular risk factors, concurrent or subsequent treatment with chemotherapy and larger total volume of the irradiated brain (Merchant et al., 2009). Long-term memory impairment is associated with increased radiation exposure to the bilateral hippocampus (Gondi et al., 2013). Delayed toxicity from RT may occur years after treatment and include severe, irreversible memory loss (Sheline et al., 1980). It is, therefore, necessary to monitor the cognitive function and daily functioning of those patients who receive RT in the long term and in the immediate post-treatment period (Pendergrass et al., 2018).

Reducing the risk of cognitive difficulties is possible by using less invasive irradiation techniques (e.g., limited fractional dose or stereotactic RT instead of WBRT) and sparing the hippocampus during irradiation (Brown et al., 2016; Okoukoni et al., 2017). In addition, proton radiotherapy, which reduces the input dose and eliminates the output dose, is also expected to contribute to the more effective preservation of cognitive function by sparing normal tissue to a greater extent (Sherman et al., 2016). Nevertheless, it is accepted that the precise reporting of the extent of cognitive changes induced by RT is difficult, and knowledge of which parts of the brain should be spared to prevent cognitive impairment is modest and requires further research (Haldbo-Classen et al., 2019).

Independent of the ongoing research into the development of more effective standard causal treatments, supportive treatment plays an important role in the management of brain tumour patients, aimed at controlling symptoms and side effects directly or indirectly caused by the tumour. Adjuvant treatments used in this group of patients include the following adverse reactions: brain oedema around the tumour site, venous thromboembolism (VTE), seizures, depression and opportunistic infections.

The standard treatment for patients with oedema of vascular origin in brain tumours is corticosteroids (e.g., dexamethasone, methylprednisolone; Gomes et al., 2005). Their long-term use may cause a variety of side effects, such as uncontrolled weight gain, Cushing’s syndrome, hyperglycaemia or diabetes, myopathy, increased susceptibility to infections (especially *Pneumocystis jiroveci* pneumonia), osteoporosis, psychiatric disorders and adrenal insufficiency. In turn, the use of antiepileptic drugs may cause a decrease in cognitive function, which suggests caution in the selection of these preparations in the treatment process (Bosma et al., 2007).

As previous studies show, endocrine therapy can also induce cognitive impairment in cancer patients (Ahles et al., 2012; Hodgson et al., 2013). A longitudinal study of cognitive performance in breast cancer patients found that adjuvant endocrine therapy was associated with slowed processing speed and verbal memory (Pendergrass et al., 2018).

### 2.3. Cognitive rehabilitation

Cognitive rehabilitation interventions are useful for treating cognitive deficits in a variety of patient populations, e.g., those with neurological disorders including traumatic brain injury and stroke, Alzheimer’s disease and epilepsy (Gehring et al., 2008). In the context of patients with brain tumours, it is clear from previous observations that neuropsychological rehabilitation should be an integral part of care (Janda et al., 2008) and should be implemented immediately, continued in a continuous manner, carried out in multiple stages and adapted to the individual needs of the patient, depending on their clinical condition (Gehring et al., 2011; Vargo et al., 2016). The effectiveness of rehabilitation is closely related to the time of its initiation. Bartolo et al. (Zucchella et al., 2013) have shown that rehabilitation is very effective if started as early as possible after the primary treatment of patients with brain tumours (Ģiga et al., 2021). It takes advantage of the adaptability and plasticity of the brain, aiming to reactivate any regenerative capacity in the nervous system. The way of conducting the improvement should be accepted by the patient and their environment, and the intensity of the exercises should be individually adapted to each patient. Early exercise helps to avoid such complications as spasticity and the acquisition of faulty movement patterns, it also stimulates the return of lost functions.

There is little data in the literature on the possible standardised techniques for cognitive rehabilitation dedicated to specific groups of patients as well as the documented monitoring of the outcome of these interventions.

One of the few reports on the cognitive rehabilitation of patients with brain tumours concerns a patient who developed cognitive deficits after right temporal bone lobectomy (Rao and Bieliauskas, 1983). A 4-month treatment programme based on the retraining of simple cognitive skills at home and psychoeducational and compensatory techniques taught in joint sessions was applied in this case. It was noted that the improvement was partly due to the effects of practice but significant progress in other tests could be attributed to the intervention used. Improvements were also related to daily and work life. Meyers and Boake (1993) published a review of general cognitive, occupational and psychological support strategies that could be incorporated into a rehabilitation programme for patients with brain tumours. Similar results were obtained by Sherer et al. (1997) who conducted a retrospective study of cognitive function. 13 patients with malignant primary brain tumours and cognitive deficits were selected for a rehabilitation programme originally developed for survivors of traumatic brain injury; this programme was applied first in a clinical setting and then in a community setting. Outcomes were described in terms of clinician ratings of independence and productivity. Changes in these non-standardised outcomes were not statistically tested. Most patients improved in both independence and productivity, and the improvements lasted 8 months (Gehring et al., 2008).

Intervention studies in the field of cognitive rehabilitation in populations with tumours outside the CNS are characterised by different treatment approaches, methods and outcomes and, therefore, different conclusions, which raises many practical problems. There is also little research in the area of interventions to treat or prevent cognitive deficits in patients with brain tumours. One potential reason for this is, among other things, the relatively low morbidity in this area. In addition, due to the low efficacy, studies have primarily focused on identifying treatments for tumours and increasing patient survival. As disease-free survival rates increase, it becomes increasingly important to develop effective cognitive rehabilitation programmes.

The treatment of a patient after brain tumour surgery requires an interdisciplinary, multidirectional approach based on a thorough functional assessment of the patient and close collaboration between the entire treatment team. In neurological and cognitive rehabilitation, there is a fixed sequence of management stages, including diagnosis, prognosis assessment, functional assessment, rehabilitation planning and their implementation by the rehabilitation team. Rehabilitation in this sense is not limited to physical improvement but also includes other areas of rehabilitation (therapy of speech and swallowing disorders, psychological problems, ability to work, social and living issues, etc.; Vargo et al., 2016). Improving cognitive functioning in patients with brain tumours usually has a beneficial effect on associated symptoms such as fatigue and mood disorders and, conversely, the effective treatment of fatigue may have indirect benefits for cognitive functioning.

### 2.4. Clinical trials

#### 2.4.1. General aspects of clinical research issues in terms of patients’ cognitive functioning

Conducting clinical trials to understand potential cognitive impairment as a consequence of cancer-related and delayed treatment effects can help clinicians and patients make informed choices that affect survival and QOL (Correa, 2006). The ideal research design to investigate the effect of treatment would be a prospective, longitudinal, double-blind and randomised placebo-controlled study with the assessment of baseline cognitive function before treatment and long-term follow-up. As such demanding conditions cannot be met in most cases, most cognitive studies should be observational (except for intervention studies) and include appropriate control groups to help determine the effect of treatment on cognitive function (Wefel et al., 2011a).

The International Cognition and Cancer Task Force (ICCTF) strongly recommends that longitudinal studies with repeated assessments should be conducted whenever possible to monitor changes in cognitive functioning. Cross-sectional studies, only after treatment, with appropriate comparison groups may be useful for exploratory analysis, hypothesis generation and proof-of-concept trials, with the confirmation of results in longitudinal studies (Wefel et al., 2011a). In the absence of pre-treatment data, the problem of limited interpretability of results arises, as differences in groups (e.g., between patients exposed and not exposed to chemotherapy) do not always reflect changes due to chemotherapy. Studies recommend pre-treatment cognitive assessment also because, as indicated by longitudinal studies to date, approximately 20–30% of patients had lower than expected pre-treatment cognitive scores (Cull et al., 1996; Bender et al., 2006; Cimprich et al., 2010).

Preoperative assessment that it may be necessary to assess the impact of cancer and its treatment on cognitive function (Taphoorn and Klein, 2004) but may be difficult to perform in practice. In most situations, cognitive testing is not commonly performed and thus results are not available for the period before cancer diagnosis. An estimate of pre-disease intellectual capacity may be based, for example, on the patient’s years and educational attainment, activities undertaken by the patient that require specific cognitive and social competencies, or by comparing the individual’s test results to population norms.

In addition, the wide range of potential factors that may influence lower than expected cognitive performance before treatment has not yet been precisely identified. According to the researchers, cognitive impairment before treatment does not appear to be explained by factors such as depression, anxiety or fatigue (Hermelink et al., 2007). On the other hand, according to Wefel et al. (2011a), it is possible that the negative effects of cancer manifest themselves in vulnerable individuals and that they are the most vulnerable to cognitive changes associated with cancer treatment; however, this requires further research.

As many patients receive a variety of anticancer therapies (e.g., chemotherapy, radiotherapy, hormone therapy, molecular targeted therapy), the outcome of cognitive function testing is in practice the effect of the combination treatment. In theory, the efficacy of a specific treatment could be assessed, but this would require the timing of drug administration in specific groups while assessing a comparison group. Findings from clinical treatment indicate that ideal comparison groups may not exist for the type of cancer or treatment of interest (Wefel et al., 2011a).

#### 2.4.2. Organisation and study of experimental and control groups

The selection of the patient population to be studied depends on the research question posed at the outset. The exclusion criteria determine the subsequent interpretation of the causes of cognitive changes. On the one hand, they may be caused by anticancer treatment—after excluding all other conditions or drugs that could affect cognitive functioning. On the other hand, the study involves patients and controls with comorbidities, e.g., hypertension, diabetes melitus and systematically taking the prescribed medication. A significant challenge and dilemma are the group of patients currently taking selective serotonin reuptake inhibitors (SSRIs). Antidepressants and SSRIs have been shown to affect cognitive performance (Hindmarch, 2009). GPs and psychiatrists often prescribe SSRIs to patients experiencing chronic stress and severe anxiety, and who do not always meet the diagnostic criteria for depression (Wefel et al., 2011a). In addition, patients with depression or anxiety may underestimate their cognitive function, and complaints may actually indicate feelings of anxiety, depression or fatigue rather than cognitive impairment (Cull et al., 1996). It is recommended in these situations that subjective complaints about cognitive function form part of brain-specific HRQOL questionnaires, which should be used alongside objective assessments of cognitive function (Taphoorn and Klein, 2004). For patients with brain tumours located in the frontal lobe, the results of these tests may not necessarily reflect the patient’s cognitive complaints, as patients may overestimate their cognitive abilities due to impaired judgement (Taphoorn et al., 1992).

For most clinical trials, it is recommended to create several control groups—disease-specific and healthy controls (both local controls and published normative data)—that undergo the same cognitive assessments at the same time as the study group. This approach may help to determine whether cognitive impairment is present and whether the apparent changes in cognitive function are due to a practice effect (i.e., a change over time associated with habituation to the assessment rather than actual improvement) or are secondary to the cancer itself, treatment or both. In a non-randomised study, the disease-specific group is likely to consist of patients with the same cancer who are receiving a different anti-cancer treatment (Wefel et al., 2011a).

It is not easy to collect a sufficient number of patients for the study to provide a basis for reliable statistical analysis and a comparison of the treatment regimens. One of the proposed solutions is to conduct the study within several institutions, where employed neuropsychologists would control the quality of procedures and data analysis.

Studies can also be conducted in the context of cooperative group studies. In this approach, a large number of patients who meet similar inclusion and exclusion criteria are randomised to a standardised treatment. A weakness of this approach is the risk of maintaining a low level of quality control and consistency in the conduct of neuropsychological assessments. Nevertheless, several studies of this type have been conducted as part of Radiation Therapy Oncology Group studies, and the results obtained may serve as a model for further work (Wefel et al., 2011a).

Analysing the issue of research into the cognitive functions of patients with brain tumours, it is assumed that further analysis of the pathophysiological mechanisms of cognitive deficits is necessary in order to adequately target interventions. The same is true for intervention studies which, based on preliminary indications of efficacy, using appropriate methodology and multivariate statistics, minimise the risk of errors of the first kind. Treatment and rehabilitation programmes aimed at alleviating other common symptoms of cancer, such as fatigue, may also be beneficial in treating cognitive symptoms and deficits. On the other hand, effective treatment of cognitive deficits may benefit other areas of functioning and patients’ quality of life.

## 3. Identification of cognitive assessment tools

The selection of test kits to assess the neurocognitive status in clinical trials is difficult due to competing demands for brevity and sensitivity. Some patients undergoing treatment with chemotherapy and radiotherapy show increased levels of fatigue and decreased motivation to perform tasks—especially long and complex tasks. Adequate brevity of the test reduces patients’ endurance requirements and provides time to measure other outcomes.

Short test batteries are also more economical and they keep the testing costs at a reasonable level, while longer batteries offer greater sensitivity in detecting cognitive impairments and changes. When preparing for clinical trials, researchers typically try to select test batteries from multiple cognitive domains (e.g., processing and motor speed, attention, visuospatial function, language, memory, executive function), but also those that are sensitive to generalised dysfunction. This is an attempt to detect potential focal changes due to the effect of the cancer as well as to detect more generalised dysfunction that may be due to drugs or other factors. The selection of appropriate tests for clinical trials is further complicated by the heterogeneous patient population with varying tumour extent and locations and, therefore, associated cognitive symptoms (Lageman et al., 2010).

In practice, brief mental status assessments such as the Mini-Mental State Examination (MMSE) are often available. However, these are considered problematic in the investigation of brain tumour patients as they only detect *delirium* or significant dementia. The MMSE has also been found to have very low sensitivity in detecting neurocognitive problems in patients with brain tumours. Self-reporting of cognitive problems (e.g., using questionnaires) is also controversial. Because of cognitive impairment, patients often rate their cognitive problems as worse. Furthermore, as studies indicate, in other cancer patient populations, self-reported cognitive impairment correlates much more strongly with fatigue and depression than with tested cognitive parameters (Meyers and Brown, 2006).

According to Meyers and Brown (2006), a battery of neurocognitive tests that would be useful in clinical research should meet the following criteria: (1) adequate brevity to reduce the burden on the patient and clinician; (2) the clinician’s possession and use of alternative forms of the test to perform the tests in a reproducible manner without compromising the results to the effect of practice; (3) adequate psychometric properties of the tool to capture significant changes in functioning in the study to the exclusion of contextual, situational factors; (4) selection of tests so that the set is sensitive to cognitive changes (care should be taken to select tests with sensitivity at the lower end of the impairment range, so that any cognitive deterioration below baseline is evident in control tests (Taphoorn and Klein, 2004); (5) high standardisation and ease of administration of the test, allowing non-neuropsychologists to learn how to use the tool; (6) including in the test kit the ability to assess cognitive function potentially damaged by tumour and treatment (e.g., assessment of frontal-subcortical function, which is often affected by radiotherapy). It is important that tests are completed by the majority of patients, even those with significant cognitive problems. This reduces the risk of selection to only well-functioning patients.

There are a few studies that examine cognitive assessment tools that have been described in systematic reviews. Most of these focus on describing which tools are most commonly used to assess people with a brain tumour diagnosis over the age of 18 years. The cognitive functions most commonly included in studies of patients with brain tumours include memory and learning, working memory for visual and auditory modalities, verbal fluency, processing speed and executive functions (Ritchie et al., 2001; Meyers and Brown, 2006; Gehring et al., 2008; Brown et al., 2013; Pendergrass et al., 2018; Haldbo-Classen et al., 2019).

Taphoorn and Klein (2004) conducted an attempt to hierarchically individualise neuropsychological testing in patients with low-grade gliomas (Klein et al., 2002) and high-grade gliomas (Klein et al., 2001) which take approximately 1 h to complete. The range of cognitive functions covered: perception or processing of information (line crossing test, Schenkenberg et al., 1980), Benton face recognition test (Benton and Van Allen, 1968) orientation test, digit symbols—Wechsler WAIS-R scale subtest, memory (Rey test of auditory-verbal learning), attention and speed of information processing (Stroop test, Categorical word fluency task, Pathfinding test, Lezak, 1995) assessment of pre-disease intellectual functioning [Adult Reading Test (Nelson HE). The national adult reading test (NART): test manual. Windsor, United Kingdom: NFER-Nelson, 1982], which relies on the current scores of reading ability or vocabulary knowledge to assess pre-disease ability. Both reading ability and vocabulary tend to be less affected by brain damage than other cognitive abilities. By using these abilities to estimate IQ, the researcher can estimate the lower limit of premorbid IQ.

Wefel et al. (2010) constructed a battery of tests for patients with brain tumours, including LGG patients, comprising Digit Span, Digit Symbol, Block Design and Similarities of the WAIS III, Trail Making Test A/B, Hopkins Verbal Learning Test, Grip Strength, Grooved Pegboard and a multilingual aphasia study consisting of the Boston Naming Test, Token Test and Controlled Oral Word Association Test (Meyers and Brown, 2006).

Another study (Haldbo-Classen et al., 2019) analysed the remote effect (approximately 7.5 years) of radiotherapy for primary brain tumours on patients’ cognitive function. The duration of the study was within 60 min; in addition, patients were given self-report questionnaires, which they completed independently for approximately 30 min. The cognitive functions examined included processing speed (Trail Making Test part A), WAIS-Coding, Stroop Interference Test), attention and working memory [Paced Auditory Serial Addition Test (PASAT), Digit Span with Wechsler Adult Intelligence Scale Version IV (WAIS-IV)], verbal learning and memory (HVLT), verbal fluency [Controlled Oral Word Association Test (COWAT)] and executive functions (Trail Making Test Part B, Stroop Interference Test).

Comparing multiple studies shows that many different tests are currently used to measure the same functions, which, in the context of conducting reliable clinical trials or rehabilitation, introduces a number of complications and inaccuracies.

312 articles on cognitive function testing in patients diagnosed with glioma were analysed by Van Coevorden-van Loon et al. (2015). A total of 46 tools for measuring cognitive function were identified, of which 5 were used more than five times. The variation was not only in the testing tools used but also in the definitions of cognitive impairment itself, which may have influenced a wide range of 19–83%. This review showed that there is no consensus on how to assess cognitive functioning in patients diagnosed with brain tumours, and that a wide range of neuropsychological tests along with different criteria are used to define cognitive dysfunction.

In an effort to understand how cancer and its treatment interact with adult patients’ cognitive-behavioural functions, the International Cognition and Cancer Task Force (ICCTF) was established in 2006 to study cancer patients’ congenital disorders (Vardy et al., 2008). This team concluded that “objective neuropsychological testing remains the gold standard for measuring cognitive function” in assessing the cognitive effects of cancer and oncological treatment. Two working groups were organised and published recommendations for common criteria for defining cognitive impairment and cognitive change, as well as specific suggestions for a core set of cognitive tests to be used to assess cognitive function in people with cancer (Pendergrass et al., 2018).

The ICCTF has also identified recommended cognitive assessment tools depending on the cognitive area of interest to the researcher. In the area of Learning and memory, the recommended tool is the Hopkins Verbal Learning Test-Revised (HVLT-R) and similar tools include the California Verbal Learning Test—II (CVLT-II), Rey Auditory Verbal Learning Test (RAVT), Brief Visuospatial Memory-Revised (BVMT-R, Visual Learning and Memory). The tasks performed by the patient consist of learning from a list of concepts, immediate and deferred recall, and recognition. For executive function, the recommended tools are Trail Making Test and Controlled Oral Word Association (COWA), as an alternative the Delis-Kaplan Executive Function Scale (D-KEFS) Trail Making or Delis-Kaplan Executive Function Scale (D-KEFS) Verbal Fluency is suggested. Tasks in this area examine a range of cognitive skills involved in performing tasks relating to attention, working memory, speed of information processing and mental flexibility as well as spontaneous word generation. Processing speed according to the ICCTF should be tested using the Trail Making Test or alternatively the Delis-Kaplan Executive Function Scale (D-KEFS) Trail Making, Wechsler Adult Intelligence Scale-IV (WAIS-IV) Coding, Symbol Digit Modalities Test (SDMT), while the cognitive skills tested here include Multiple cognitive skills involved in performing a task, including attention, working memory, information processing speed, and mental flexibility. Working memory is recommended to be measured by the Auditory Consonant Trigrams (ACT; tested functions are short term or working memory task requiring on-line maintenance of information while performing an interference task during a delay), Paced Auditory Serial Attention Test (PASAT; functions: Serial attention task assessing working memory, divided attention, and information processing speed), Brief Test of Attention (BTA; Auditory divided attention), and Wechsler Adult Intelligence Scale -IV (WAIS-IV) Letter Number Sequencing (functions: working memory, attention and mental control).

There is a need to systematise cognitive testing tools. Currently, there is a wide variety of tests measuring the same or similar cognitive parameters. Tests should cover many areas, but this depends on the type of research being conducted, e.g., treatment process, cognitive rehabilitation, clinical trials with experimental and control groups.

## 4. Use of cognitive assessment tools to evaluate functional disability

In addition to the need for the reliable assessment of cognitive function in patients with brain tumours, their rehabilitation and the consequent need for functional disability assessment is an important issue (Ģiga et al., 2021). There are currently no standardised neuropsychological tools for the functional disability assessment of people with brain tumours. The International Classification of Functioning, Disability and Health (ICF), which provides a common standard language for identifying disability and also offers a framework for coding health information on a large scale, can provide a basis for their development, use and comparison. By combining information on clinical diagnosis according to the widely used ICD-11 classification and information from the ICF classification of functioning, a broader and more meaningful picture of the health of individuals and populations can be achieved, which would inform decision-making in the broad field of health promotion.

The International Classification of Functioning, Disability and Health (ICF) was introduced by the WHO in 2001 as a comprehensive coding system for functioning and disability. It creates a new conceptual framework for disability and provides a “common language” for all professions. Since then, the classification has continued to be improved and disseminated, and more countries have signed a convention to implement it. The ICD-10 and the ICF are complementary and users are encouraged to use them together: the ICD-10 provides a ‘diagnosis’ of diseases, disorders or other health conditions and the ICF complements this information in terms of the functioning of the individual. The ICF can also serve as an epidemiological tool and allow the collection and comparison of disability data from different countries with different legislation, definitions of disability, standards of living and welfare policies.

**Figure 1:**
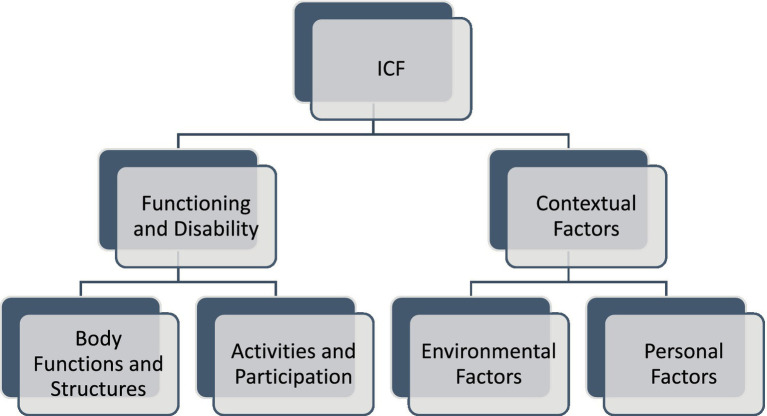
The ICF classification of health and health-related conditions for children and adult for interprofessional collaborative practice and person-centred care. The framework consists of “Functioning and Disability” and “Contextual Factors.” “Functioning and disability” is characterised by “Body functions and structures” and “Activity and participation” while “Contextual factors” refer to the environment in which a person lives. Body Functions and Structures describes actual anatomy and physiology/psychology of the human body. Activity and participation describes the person’s functional status, including communication, mobility, interpersonal interactions, self-care, learning, applying knowledge etc. *Environmental Factors* include factors that are not within the person’s control, such as family, work, government agencies, laws and cultural beliefs. *Personal Factors* include race, gender, age, educational level, coping styles etc. (Stucki, 2005).

**Figure 2:**
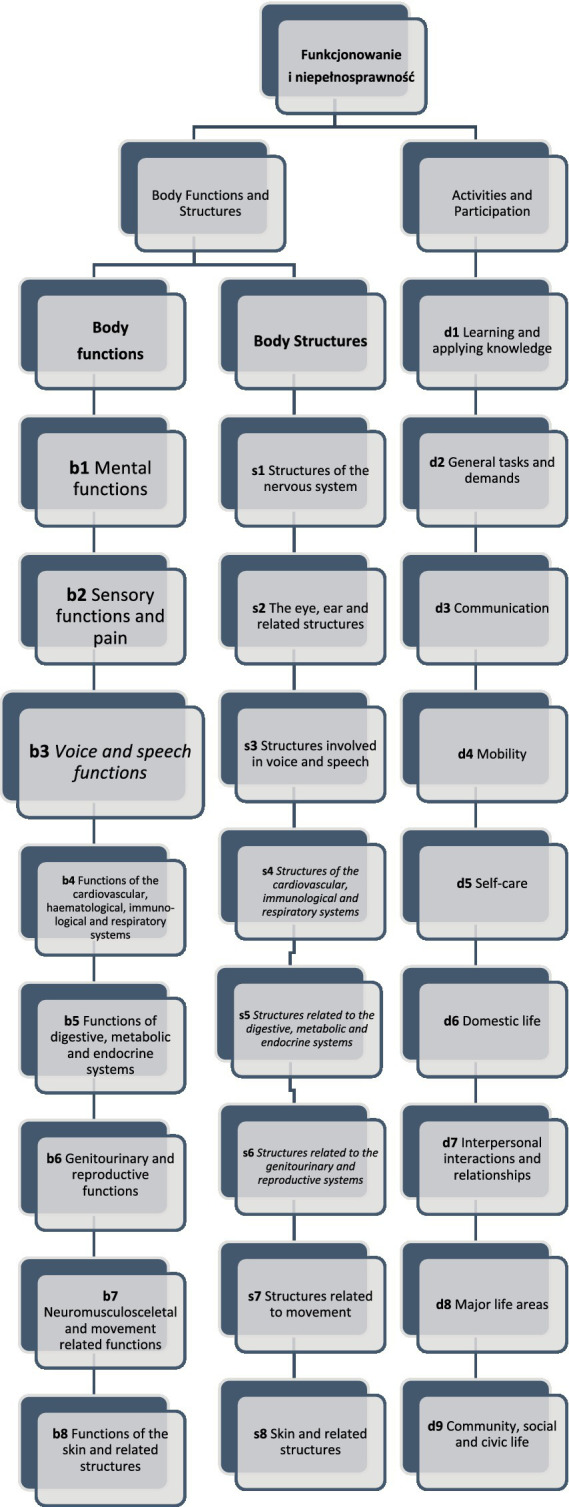
The functioning and disability framework of the ICF classification. Different parts of body functions and body structures correspond to each other and have been arranged according to the systems of the human body. The field “Activity and participation” describes the person’s current tasks or activities in the most important areas of daily life like learning. (Stucki, 2005; Bickenbach et al., 2012; WHO, 2020).

**Figure 3:**
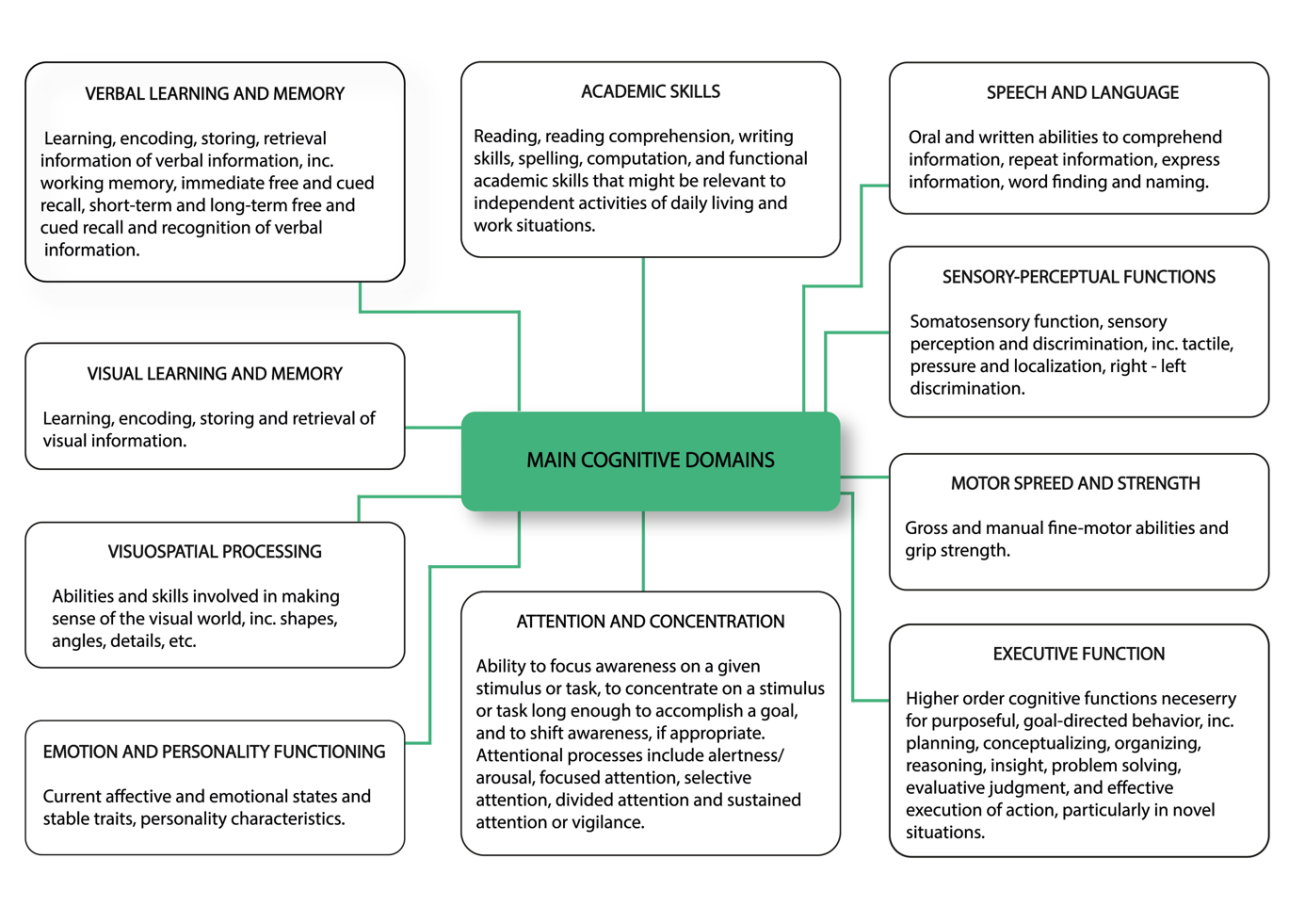
Characteristics of basic cognitive functions for neuropsychological evaluation of cancer patients. The proposed domains include learning and memory, visual learning and memory, visuospatial processing, emotion and personality functioning, academic skills, speech and language, sensory and language, sensory-perceptual functions, motor speed and strength, executive functions as well as attention and concentration (Pendergrass et al., 2018, changed).

**Figure 4:**
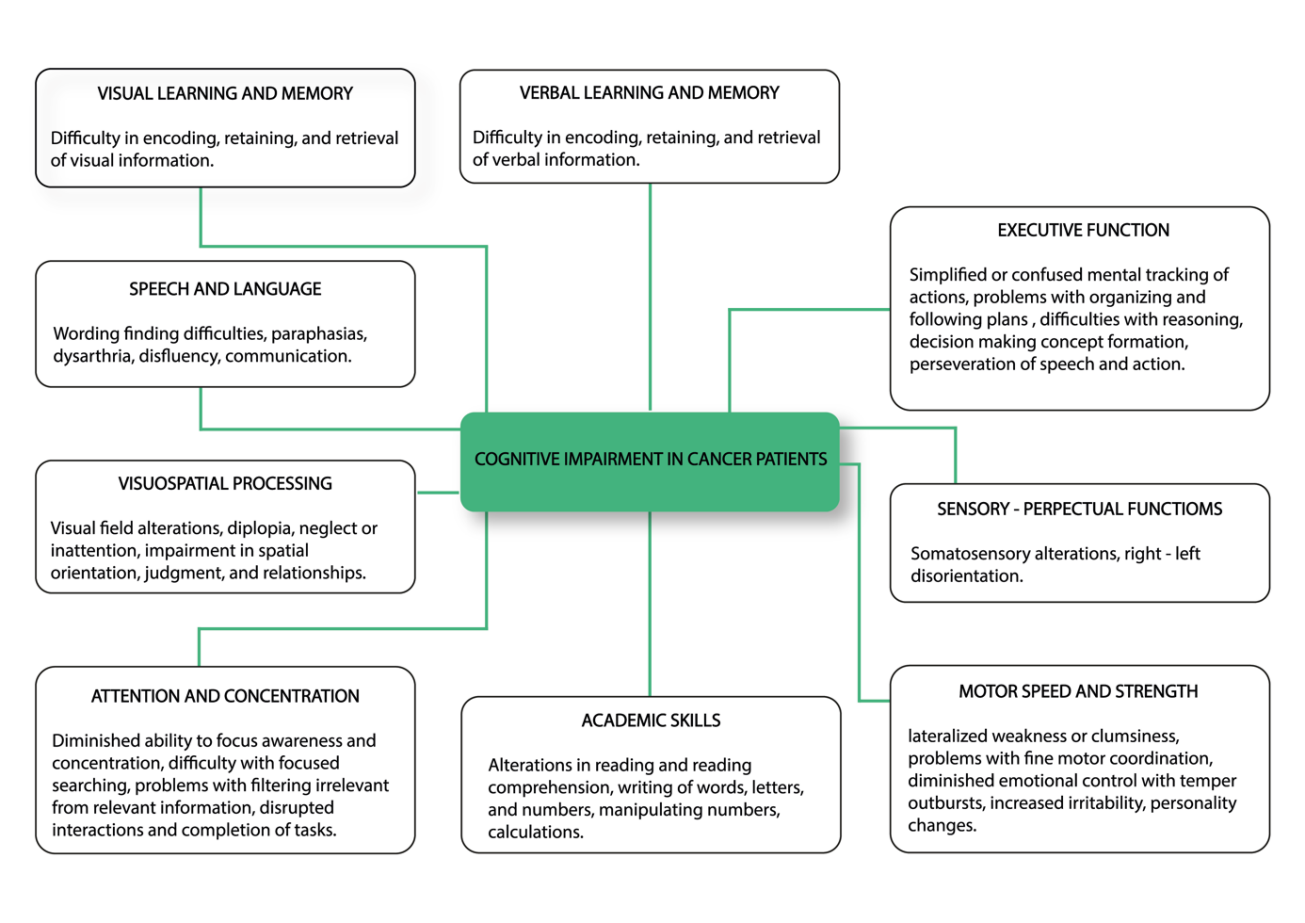
Common cognitive impairments identified in cancer patients including visual learning and memory, speech and language, visuospatial processing, attention and concentration, academic skills, motor speed and strength, sensory-perceptual functions and executive function (Pendergrass et al., 2018, changed).

The ICF consists of two parts—the first is “Functioning and Disability” and the second describes “Contextual Factors” (Pendergrass et al., 2018). “Functioning and disability” are characterised by “Body functions and structures” and “Activity and participation” while “Contextual factors” refer to the environment in which a person lives (Figure 1). The different parts of structures and functions correspond to each other and have been arranged according to the systems of the human body (Figure 2). The field “Activity and participation” describes the person’s current tasks or activities in the most important areas of daily life, e.g., learning and expanding one’s competences, communication, mobility, independence in terms of self-care and looking after one’s own needs, functioning in private and professional space, ability to initiate and maintain social interaction. Participation itself is defined as the involvement of the person in certain life situations, which widens the scope of assessment to include the motivational aspect. Any difficulties that prevent action are called activity limitations in the ICF classification, while problems that make it difficult for the person to engage in life situations are called participation limitations. The characteristics of basic cognitive functions for the neuropsychological evaluation of cancer patients are described in Figure 3. The proposed domains include learning and memory, visual learning and memory, visuospatial processing, emotion and personality functioning, academic skills, speech and language, sensory and language, sensory-perceptual functions, motor speed and strength, executive functions, and attention and concentration. Within the performance of individual functions in cancer patients, specific difficulties related to their impairment can be identified (Figure 4).

Table 1 compares the commonly used tools for cognitive diagnosis of patients with brain tumours according to selected ICF components (Klein et al., 2001; Brown et al., 2003; Ek et al., 2005; Laack et al., 2005; Meyers and Brown, 2006; Bosma et al., 2007; Gehring et al., 2008; Douw et al., 2009; Ruge et al., 2011; Blonski et al., 2012; Santini et al., 2012; Van Coevorden-van Loon et al., 2015; Pendergrass et al., 2018; Haldbo-Classen et al., 2019; Ģiga et al., 2021). In this way, areas that can be used to assess the functional disability of patients with brain tumours were identified. Taking into account the specificity of the functioning of patients with a brain tumour diagnosis, mental functions (b1) were included in the scope of the ICF criteria for bodily functions, and included: functions of consciousness, orientation and intellectual functions, temperament and personality functions, attention and memory functions, psychomotor, emotional, perceptual, thinking, language functions, computational skills and functions of sequencing complex movements. Activity and participation took into account the ability to learn and apply knowledge (d1), general tasks and requirements for daily functioning and task taking (d2), and communicative functions (d3).

**Table 1:** The comparison of selected tools for cognitive diagnosis of patients with brain tumours with ICF components.

Components	BN-20	C30	MMSE	ACE-III	TMT	CTT	CVLT	WCST	BENTON	STROOP
*Cognitive functions*										
*b1 Mental functions*										
b110 Awareness functions	•									
b114 Guidance functions			•	•						
b117 Intellectual functions					•	•		•		
b126 Temperament and personality functions		•								
b140 Note functions		•	•	•	•	•	•	•	•	•
b144 Memory functions		•	•	•			•	•	•	
b147 Features psychomotor				•	•	•				
b152 Emotional functions		•								
b156 Perceptual functions	•		•	•	•	•			•	
b160 Thought functions			•	•	•	•		•		
b167 Mental functions of language	•		•	•			•			
b172 Calculation functions			•	•						
b176 Mental functions of sequencing complex movements			•	•						
*Activities and Participation*										
*d1 Learning and applying knowledge*										
d110 Watching			•	•	•					
d115 Listening			•	•	•					
d160 Focusing attention			•	•	•					
d163 Thinking				•	•					
d166 Reading	•	•	•	•	•					
*d2 General tasks and requirements*										
d220 Multi-tasking					•					
d230 Carrying out daily activities	•				•					
*d3 Communication*										
d310 Communicating with receiving spoken messages			•							
d315 Communicating with receiving non-verbal messages					•					
d330 Speaking		•	•	•	•					
d345 Writing messages			•	•	•					

These tools include tests for evaluation of cognitive functions (MMSE, ACE-III, TMT A and B, CTT, CVLT, WSCT; Benton and Stroop), and questionnaires for subjective self-assessment of the quality of life (EORTC QLQ-C30 and EORTC QLQ- BN20). The following areas were identified and included in the ICF criteria: mental functions, b1 (functions of awareness, orientation and intellectual functions, temperament and personality functions, attention and memory functions, psychomotor, emotional, perceptual functions, mental, linguistic, computational and sequencing functions of complex movements), activity and participation, d1 (the ability to learn and apply knowledge), general tasks and requirements for daily functioning and task performance (d2) and communication functions (d3).

In terms of the tools used to objectively measure cognitive function, they included: MMSE, ACE-III, TMT A and B, CTT, CVLT, WSCT; Benton test, Stroop test, while among the tools for subjective self-assessment regarding quality of life, EORTC QLQ-C30 and EORTC QLQ- BN20 were selected. Among the neuropsychological assessment tools, the MMSE and TMT are among the most commonly used in the diagnosis of people with brain tumours, with the psychometric properties of the MMSE being better documented (Ģiga et al., 2021). In the following, a brief description of the above tools is presented with a particular emphasis on their practical application in measuring specific variables.

*EORTC QLQ-C30 questionnaire* contains five scales assessing functional status, relating to: physical functioning, social role performance, emotional functioning, memory and concentration, social functioning as well as three scales assessing disease symptoms: fatigue, nausea, vomiting and pain as well as an overall health/quality of life rating scale. In addition, it includes six single questions assessing illness symptoms such as loss of appetite, shortness of breath, insomnia, constipation, diarrhoea and financial difficulties as a consequence of illness (Aaronson et al., 1993).

*EORTC QLQ- BN20 questionnaire* addresses the impact and treatment of brain tumours on patients’ symptoms, functioning and health-related quality of life in both clinical trials and practice. The questionnaire was originally used for patients with glioma, but over the years there has been an increase in the use of this questionnaire among patients with other brain tumours, both primary and metastatic. The tool consists of four multi-item scales that relate to: future uncertainty; visual impairment; motor dysfunction; and communication deficit. In addition, seven individual items assess headache, seizures, drowsiness, hair loss, itchy skin, leg weakness and bladder control. All of the items and scores are assessed on a 0–100 scale, with higher scores reflecting more severe symptoms.

The findings confirmed the relevance and reliability of the questionnaire (Taphoorn et al., 2010).

*MMSE—Mini Mental State Examination—Mini-Mental* is a clinical scale used to measure impairments in a patient’s cognitive functioning. It consists of 30 questions/tasks allowing for a quantitative assessment of various aspects of cognitive functioning. The areas assessed include: orientation in time and place, remembering, attention and counting, recalling, naming, repeating, understanding, reading, writing and drawing. It also allows for the assessment of the sequencing of complex movements. It has high internal consistency verified for the entire clinical sample of individual patient groups as well as high diagnostic accuracy confirmed by the method of intergroup comparisons. The test is mainly used in the screening of cognitive functions (mainly dementia processes) and monitoring of the course of the disease. However, it is indicated that it should not be used as a stand-alone diagnostic tool to identify dementia (Tombaugh and Mclntyre, 1992).

*Addenbrooke’s Cognitive Examination-III (ACE-III)* is a test that assesses five core areas of cognitive functioning: attention, memory, verbal fluency, language function, and visuospatial function. Tasks assess orientation functions, perception, thinking, computational functions and sequencing of movements. The test is a new version of Addenbrooke’s Cognitive Examination-Revised (ACE-R). The overall test score ranges from 0 to 100, with higher scores indicating better cognitive functioning. The ACE-III takes an average of 15 min to complete, which is important in the context of testing patients who exhibit elevated levels of fatigue (Hsieh et al., 2013). The ACE-III scale can be used in clinical practice by both psychologists and medical specialists (mainly neurologists, geriatricians, psychiatrists). The ACE-III test can also be carried out by appropriately trained other medical staff (e.g., nurses), although the results can only be interpreted by a psychologist or specialist physician with clinical experience in dementias. The primary use of the ACE-III scale is for the early detection of primary degenerative brain diseases (Lonie et al., 2010) and differentiating them from mental illness (Hornberger et al., 2009). The scale can also be used to monitor disease progression (Kipps et al., 2008).

*Trail Making Test/Test of Connecting Points A and B (TMT A and B)* assesses visuospatial aspects of working memory, general attentional efficiency, search-field functions, thinking functions and in particular interhemispheric functioning. The TMT consists of two parts: in part A, the patient combines consecutive numbers; in part B, the patient alternates between consecutive numbers and letters of the alphabet. The result of the tests is the time needed to draw the path correctly. If the time to complete TMT B is twice as long as the time to complete TMT A, this is considered to indicate frontal cortex dysfunction (Zhao et al., 2013; Kim, 2021).

*CTT: Connecting Numbers and Colours Test (CTT, Color Trails Test)* is a test used to examine a variety of intellectual processes related to attention and executive functions, and in particular to assess the purposeful searching of material, sustained and metastatic attention, sequential processing of information, thinking and monitoring of one’s own behaviour. Visual-motor skills are also involved in performance. CTT is a test similar to TMT—it also consists of two parts—in the first part of the test the respondent has to connect coloured circles with numbers from 1 to 25 in the shortest possible time. In the second part of the test, the respondent is instructed to connect alternating numbers with colours (most often pink and yellow are used). The result is the execution time, the number of hints, the number of mistakes in the order of colours and numbers. The maximum time for each part of the test is 4 min. The test demonstrates high reliability in terms of absolute stability of the indices, high constancy of clinical interpretations, and confirmed diagnostic accuracy by means of intergroup comparisons, which involved people with different locations and different aetiologies of brain damage (Zhao et al., 2013).

*California Verbal Learning Test (CVLT)* is used to measure attention, the ability to learn and remember verbal material, and the mental functions of language. It consists of three lists of words: List A, List B and a list of words to be recognised. Lists A and B each contain 16 categorically related words. The recognition word list contains 44 stimuli including the whole List A, some words from List B, and other distractor words fulfilling certain conditions. A limitation of the test is its long execution time (approximately 60 min), which may be difficult for patients with increased levels of fatigue. Moreover, the CVLT has only one version and the study of the dynamics of changes in memory functioning is in its case burdened with the risk of the re-presentation of the same material. The reliability and validity of the test has been confirmed, showing proven correlations with measures of cognitive function and confirmed diagnostic accuracy using intergroup comparisons (these included individuals with different locations and different aetiologies of brain injury; Woods et al., 2006).

*Wisconsin Card Sorting Test (WCST)* is considered to be a diagnostically specific tool for assessing working memory and, in addition, for evaluating executive function disorders and problem solving skills. The performance of this type of test requires a complex pattern of functioning that takes into account instructions and goals, taking into account current experience and feedback as well as planning a specific strategy (Stratta et al., 1997). Tests of this type share a common neurophysiological dimension in the form of the prefrontal cortex region of the brain. The most important measurements in the test are considered to be the number of perseverative errors made and the number of categories correctly arranged, and the occurrence of change in the correctly detected category of card distribution is also noted (Greve et al., 1998; Greve, 2001). Correct performance requires the retention of the test purpose in memory, retention of auditory and visual attention, retained learning, categorisation and executive control, and correct thinking (Artiolai Fortuny and Heaton, 1996).

*Benton test* is used to test attention and working memory for the visual modality. The respondent draws presented patterns from memory or redraws them. Depending on the version of the test chosen, the test lasts a maximum of several minutes, although there is no time limit on the activity side for the patient to complete the task (Obayashi et al., 2003). The tool has three alternative parallel versions C, D, and E, which can be used in a test with four alternative methods (A, B, C, and D), which is useful in situations where successive tests have to be performed in small time intervals. The high reliability of the test was demonstrated for two basic indicators, i.e., the number of correct representations and the number of errors made. The high validity of the test is evidenced, among other things, by data on the decrease of the results with the age of the subjects, high correlations with other tests measuring memory and attention and executive functions, poorer performance of the test by persons with CNS damage, mild cognitive disorders as well as patients with depression and dementia (Seo et al., 2007).

*Stroop Colour-Word Interference Test* is a measure of cognitive control over the disruptive effects of an automated reading response; hence, it has also been used to measure inhibitory control in conflict situations. Depending on the paradigm adopted by the researcher regarding the relationship between the human executive and cognitive systems, the Stroop test is considered to be an indicator of working memory functioning, attention capacity or executive control. In addition to the neuropsychological assessment of patients with focal brain damage and degenerative CNS diseases, the Stroop test is also used in the diagnosis of patients with various psychiatric disorders as well as the impact of the aging process on the ability to inhibit responses in healthy individuals (Stuss et al., 2001).

## 5. Conclusion

Cognitive impairment is characteristic of most patients with a diagnosis of a malignant brain tumour. It results not only from the consequences of the tumour itself, but also from treatment-induced neurotoxicity. In addition, cognitive impairment can be divided into transient cognitive impairment, occurring immediately before and during treatment as well as persistent cognitive deficits that persist long after treatment, resulting from disseminated brain changes. Currently, there are limited number of standardised, universal procedures for neuropsychological monitoring of patients with brain tumour diagnosis, which seems to be of key importance not only for the implementation of the treatment process itself, but also for the improvement of patients’ quality of life. This article points to the need to systematise research tools or develop new ones, adapted to diagnostic needs with high psychometric characteristics, with particular attention to memory processes and learning effect. Rehabilitation of patients is also an important issue, which requires the use of adequate tools to assess functional disability. The International Classification of Functioning, Disability and Health (ICF) seems to be useful in this respect. The ICF has the advantage of targeting actions to improve the condition of the individual and to keep them as long as possible in a state of well-being that allows them to function effectively in society or to return to work. This is particularly important in view of the ageing population and the increasing number of diagnoses related to brain tumours.

## 6. Limitations

The article can be useful for summarizing the literature. However, the nature of any narrative review is subjective in the determination of which studies to include, the way the studies are analyzed, and the conclusions drawn. There is possibility of misleading in drawing conclusions that are normally due to selection bias, subjective weighing of the studies chosen for the review, unspecified inclusion criteria, and failure to consider the relationships between study characteristics and study results.

## Author contributions

AgP has given substantial contributions to the conception or the design of the manuscript. KH and AnP to acquisition and interpretation of the data. AgP, AnP, and KH have participated to drafting the manuscript. KH revised the manuscript critically. All authors have read and approved the final version of the manuscript.

## Funding

This research was funded by Grant NCN-OPUS 19: UMO-2020/37/B/NZ7/01122.

## Conflict of interest

The authors declare that the research was conducted in the absence of any commercial or financial relationships that could be construed as a potential conflict of interest.

## Publisher’s note

All claims expressed in this article are solely those of the authors and do not necessarily represent those of their affiliated organizations, or those of the publisher, the editors and the reviewers. Any product that may be evaluated in this article, or claim that may be made by its manufacturer, is not guaranteed or endorsed by the publisher.

## References

[ref1] Aaronson N. K. Ahmedzai S. Bergman B. Bullinger M. Cull A. Duez N. J.. (1993). The European Organization for Research and Treatment of cancer QLQ-C30: a quality-of-life instrument for use in international clinical trials in oncology. J. Natl. Cancer Inst. 85, 365–376. doi: 10.1093/jnci/85.5.365, PMID: 8433390

[ref2] Ahles T. A. Root J. C. Ryan E. L. (2012). Cancer- and cancer treatment-associated cognitive change: an update on the state of the science. J. Clin. Oncol. 30, 3675–3686. doi: 10.1200/JCO.2012.43.0116, PMID: 23008308 PMC3675678

[ref3] Ahles T. A. Saykin A. J. McDonald B. C. Li Y. Furstenberg C. T. Hanscom B. S.. (2010). Longitudinal assessment of cognitive changes associated with adjuvant treatment for breast cancer: impact of age and cognitive reserve. J. Clin. Oncol. 28, 4434–4440. doi: 10.1200/JCO.2009.27.0827, PMID: 20837957 PMC2988635

[ref4] Artiolai Fortuny L. Heaton R. K. (1996). Standard versus computerized administration of the Wisconsin card sorting test. Clin. Neuropsychol. 10, 419–424. doi: 10.1080/13854049608406702

[ref5] Bender C. M. Sereika S. M. Berga S. L. Vogel V. G. Brufsky A. M. Paraska K. K.. (2006). Cognitive impairment associated with adjuvant therapy in breast cancer. Psycho-Oncology 15, 422–430. doi: 10.1002/pon.964 16097037

[ref6] Benton A. L. Van Allen M. W. (1968). Impairment in facial recognition in patients with cerebral disease. Trans. Am. Neurol. Assoc. 4, 344–IN1. doi: 10.1016/s0010-9452(68)80018-8 5711050

[ref7] Beuriat P. A. Cristofori I. Gordon B. Grafman J. (2022). The shifting role of the cerebellum in executive, emotional and social processing across the lifespan. Behav. Brain Funct. 18:6. doi: 10.1186/s12993-022-00193-5, PMID: 35484543 PMC9047369

[ref1001] Bickenbach J. Cieza S. Rauch S. Stucki G. (2012). ICF Core Sets Manual for Clinical Practice. Göttingen, Germany: Hogrefe.

[ref8] Björklund A.-C. Granlund M. Santacroce S. J. Enskär K. Carlstein S. Björk M. (2021). Using ICF to describe problems with functioning in everyday life for children who completed treatment for Brain tumor: an analysis based on professionals’ documentation. Front. Rehabil. Sci. 2, 708265. doi: 10.3389/fresc.2021.708265, PMID: 36188761 PMC9397836

[ref9] Blonski M. Taillandier L. Herbet G. Maldonado I. L. Beauchesne P. Fabbro M.. (2012). Combination of neoadjuvant chemotherapy followed by surgical resection as a new strategy for WHO grade II gliomas: a study of cognitive status and quality of life. J. Neuro-Oncol. 106, 353–366. doi: 10.1007/s11060-011-0670-x, PMID: 21785913

[ref10] Bosma I. Vos M. J. Heimans J. J. Taphoorn M. J. B. Aaronson N. K. Postma T. J.. (2007). The course of neurocognitive functioning in high-grade glioma patients. Neuro-Oncology 9, 53–62. doi: 10.1215/15228517-2006-012, PMID: 17018697 PMC1828106

[ref11] Brain Tumor Progress Review Group, National Cancer Institute (U.S.) and National Institute of Neurological Disorders and Stroke (U.S.) (2000). Report of the Brain Tumor Progress Review Group, Bethesda, MD: NIH Publication, 96.

[ref12] Brown P. D. Buckner J. C. O’Fallon J. R. Iturria N. L. Brown C. A. O’Neill B. P.. (2003). Effects of radiotherapy on cognitive function in patients with low-grade glioma measured by the Folstein mini-mental state examination. J. Clin. Oncol. 21, 2519–2524. doi: 10.1200/JCO.2003.04.172, PMID: 12829670

[ref13] Brown P. D. Jaeckle K. Ballman K. V. Farace E. Cerhan J. H. Anderson S. K.. (2016). Effect of radiosurgery alone vs radiosurgery with whole brain radiation therapy on cognitive function in patients with 1 to 3 brain metastases a randomized clinical trial. JAMA 316, 401–409. doi: 10.1001/jama.2016.9839, PMID: 27458945 PMC5313044

[ref14] Brown P. D. Pugh S. Laack N. N. Wefel J. S. Khuntia D. Meyers C.. (2013). Memantine for the prevention of cognitive. Neuro-Oncology 15, 1429–1437. doi: 10.1093/neuonc/not114, PMID: 23956241 PMC3779047

[ref15] Cieza A. Fayed N. Bickenbach J. Prodinger B. (2019). Refinements of the ICF linking rules to strengthen their potential for establishing comparability of health information. Disabil. Rehabil. 41, 574–583. doi: 10.3109/09638288.2016.1145258, PMID: 26984720

[ref16] Cimprich B. Reuter-Lorenz P. Nelson J. Clark P. M. Therrien B. Normolle D.. (2010). Prechemotherapy alterations in brain function in women with breast cancer. J. Clin. Exp. Neuropsychol. 32, 324–331. doi: 10.1080/13803390903032537, PMID: 19642048

[ref17] Coomans M. B. van der Linden S. D. Gehring K. Taphoorn M. J. B. (2019). Treatment of cognitive deficits in brain tumour patients: current status and future directions. Curr. Opin. Oncol. 31, 540–547. doi: 10.1097/cco.0000000000000581, PMID: 31483326 PMC6824580

[ref18] Correa D. D. (2006). Cognitive functions in Brain tumor patients. Hematol. Oncol. Clin. North Am. 20, 1363–1376. doi: 10.1016/j.hoc.2006.09.012 17113468

[ref19] Correa D. D. Maron L. Harder H. Klein M. Armstrong C. L. Calabrese P.. (2007). Cognitive functions in primary central nervous system lymphoma: literature review and assessment guidelines. Ann. Oncol. 18, 1145–1151. doi: 10.1093/annonc/mdl464, PMID: 17284616

[ref20] Corry J. Smith J. G. Wirth A. Quong G. Liew K. H. (1998). Primary central nervous system lymphoma: age and performance status are more important than treatment modality. Int. J. Radiat. Oncol. Biol. Phys. 41, 615–620. doi: 10.1016/S0360-3016(97)00571-3, PMID: 9635710

[ref21] Cull A. Hay C. Love S. B. Mackie M. Smets E. Stewart M. (1996). What do cancer patients mean when they complain of concentration and memory problems? Br. J. Cancer 74, 1674–1679. doi: 10.1038/bjc.1996.608, PMID: 8932354 PMC2074867

[ref22] D’Souza S. Hirt L. Ormond D. R. Thompson J. A. (2021). Retrospective analysis of hemispheric structural network change as a function of location and size of glioma. Brain Commun. 3, 216. doi: 10.1093/braincomms/fcaa216, PMID: 33501423 PMC7811759

[ref23] Damasio H. Tranel D. Anderson S. W. (1990). Neuropsychological impairments associated with lesions caused by tumor or stroke. Arch. Neurol. 47, 397–405. doi: 10.1001/archneur.1990.00530040039017 2322133

[ref24] Douw L. Klein M. Fagel S. S. A. A. van den Heuvel J. Taphoorn M. J. B. Aaronson N. K.. (2009). Cognitive and radiological effects of radiotherapy in patients with low-grade glioma: long-term follow-up. Lancet Neurol. 8, 810–818. doi: 10.1016/S1474-4422(09)70204-2, PMID: 19665931

[ref25] Durand T. Bernier M. O. Léger I. Taillia H. Noël G. Psimaras D.. (2015). Cognitive outcome after radiotherapy in brain tumor. Curr. Opin. Oncol. 27, 510–515. doi: 10.1097/CCO.0000000000000227 26371778

[ref26] Ek L. Smits A. Påhlson A. Almkvist O. (2005). Analysis of cognitive dysfunction in patients with low-grade glioma. J. Clin. Psychol. Med. Settings 12, 165–173. doi: 10.1007/s10880-005-3276-7

[ref27] Gehring K. Aaronson N. K. Taphoorn M. J. B. Sitskoorn M. M. (2011). A description of a cognitive rehabilitation programme evaluated in brain tumour patients with mild to moderate cognitive deficits. Clin. Rehabil. 25, 675–692. doi: 10.1177/0269215510395791, PMID: 21421690

[ref28] Gehring K. Sitskoorn M. M. Aaronson N. K. Taphoorn M. J. B. (2008). Interventions for cognitive deficits in adults with brain tumours. Lancet Neurol. 7, 548–560. doi: 10.1016/S1474-4422(08)70111-X 18485318

[ref29] Ģiga L. Pētersone A. Čakstiņa S. Bērziņa G. (2021). Comparison of content and psychometric properties for assessment tools used for brain tumor patients: a scoping review. Health Qual. Life Outcomes 19:234. doi: 10.1186/s12955-021-01863-0, PMID: 34625062 PMC8501604

[ref30] Gomes J. A. Stevens R. D. Lewin J. J. III Mirski M. A. Bhardwaj A. (2005). Glucocorticoid therapy in neurologic critical care. Crit. Care Med. 33, 1214–1224. doi: 10.1097/01.CCM.0000166389.85273.38 15942333

[ref31] Gondi V. Hermann B. P. Mehta M. P. Tomé W. A. (2013). Hippocampal dosimetry predicts neurocognitive function impairment after fractionated stereotactic radiotherapy for benign or low-grade adult brain tumors. Int. J. Radiat. Oncol. Biol. Phys. 85, 348–354. doi: 10.1016/j.ijrobp.2012.11.031 23312272

[ref32] Gould J. (2018). Breaking down the epidemiology of brain cancer. Nature 561, S40–S41. doi: 10.1038/d41586-018-06704-7, PMID: 30258156

[ref33] Greve K. W. (2001). The WCST-64: a standardized short-form of the Wisconsin card sorting test. Clin. Neuropsychol. 15, 228–234. doi: 10.1076/clin.15.2.228.1901, PMID: 11528544

[ref34] Greve K. W. Ingram F. Bianchini K. J. (1998). Latent structure of the Wisconsin card sorting test in a clinical sample. Arch. Clin. Neuropsychol. 13, 597–609. doi: 10.1016/S0887-6177(97)00075-9, PMID: 14590620

[ref35] Haldbo-Classen L. Amidi A. Wu L. M. Lukacova S. Oettingen G. V. Gottrup H.. (2019). Long-term cognitive dysfunction after radiation therapy for primary brain tumors. Acta Oncol. 58, 745–752. doi: 10.1080/0284186X.2018.1557786, PMID: 30757955 PMC6644714

[ref36] Hense K. Plank T. Wendl C. Dodoo-Schittko F. Bumes E. Greenlee M. W.. (2021). fMRI retinotopic mapping in patients with brain tumors and space-occupying brain lesions in the area of the occipital lobe. Cancers 13, 2439. doi: 10.3390/cancers13102439, PMID: 34069930 PMC8157607

[ref37] Hermelink K. Untch M. Lux M. P. Kreienberg R. Beck T. Bauerfeind I.. (2007). Cognitive function during neoadjuvant chemotherapy for breast cancer: results of a prospective, multicenter, longitudinal study. Cancer 109, 1905–1913. doi: 10.1002/cncr.22610 17351951

[ref38] Hindmarch I. (2009). Cognitive toxicity of pharmacotherapeutic agents used in social anxiety disorder. Int. J. Clin. Pract. 63, 1085–1094. doi: 10.1111/j.1742-1241.2009.02085.x, PMID: 19570125

[ref39] Hodgson K. D. Hutchinson A. D. Wilson C. J. Nettelbeck T. (2013). A meta-analysis of the effects of chemotherapy on cognition in patients with cancer. Cancer Treat. Rev. 39, 297–304. doi: 10.1016/j.ctrv.2012.11.001 23219452

[ref40] Hornberger M. Shelley B. P. Kipps C. M. Piguet O. Hodges J. R. (2009). Can progressive and non-progressive behavioural variant frontotemporal dementia be distinguished at presentation? J. Neurol. Neurosurg. Psychiatry 80, 591–593. doi: 10.1136/jnnp.2008.163873, PMID: 19228667

[ref41] Hsieh S. Schubert S. Hoon C. Mioshi E. Hodges J. R. (2013). Validation of the Addenbrooke’s cognitive examination III in frontotemporal dementia and Alzheimer’s disease. Dement. Geriatr. Cogn. Disord. 36, 242–250. doi: 10.1159/000351671, PMID: 23949210

[ref42] Janda M. Steginga S. Dunn J. Langbecker D. Walker D. Eakin E. (2008). Unmet supportive care needs and interest in services among patients with a brain tumour and their carers. Patient Educ. Couns. 71, 251–258. doi: 10.1016/j.pec.2008.01.020, PMID: 18329220

[ref43] Keime-Guibert F. Napolitano M. Delattre J. Y. (1998). Neurological complications of radiotherapy and chemotherapy. J. Neurol. 245, 695–708. doi: 10.1007/s004150050271 9808237

[ref1002] Kim D. H. (2021). Rehabilitation therapy for patients with osteoporosis. J. Korean Med. Assoc. 64, 366–372. doi: 10.5124/JKMA.2021.64.5.366

[ref44] Khan F. Amatya B. (2013). Use of the international classification of functioning, disability and health (ICF) to describe patient -reported disability in primary brain tumour in an Australian comunity cohort. J. Rehabil. Med. 45, 434–445. doi: 10.2340/16501977-1132, PMID: 23584801

[ref45] Kipps C. M. Nestor P. J. Dawson C. E. Mitchell J. Hodges J. R. (2008). Measuring progression in frontotemporal dementia: implications for therapeutic interventions. Neurology 70, 2046–2052. doi: 10.1212/01.wnl.0000313366.76973.8a 18505978

[ref46] Klein M. Heimans J. J. Aaronson N. K. van der Ploeg H. Grit J. Muller M.. (2002). Effect of radiotherapy and other treatment-related factors on mid-term to long-term cognitive sequelae in low-grade gliomas: a comparative study. Lancet 360, 1361–1368. doi: 10.1016/S0140-6736(02)11398-5, PMID: 12423981

[ref47] Klein M. Taphoorn M. J. B. Heimans J. J. van der Ploeg H. M. Vandertop W. P. Smit E. F.. (2001). Neurobehavioral status and health-related quality of life in newly diagnosed high-grade glioma patients. J. Clin. Oncol. 19, 4037–4047. doi: 10.1200/JCO.2001.19.20.4037, PMID: 11600605

[ref48] Laack N. N. Brown P. D. Ivnik R. J. Furth A. F. Ballman K. V. Hammack J. E.. (2005). Cognitive function after radiotherapy for supratentorial low-grade glioma: a north central cancer treatment group prospective study. Int. J. Radiat. Oncol. Biol. Phys. 63, 1175–1183. doi: 10.1016/j.ijrobp.2005.04.016, PMID: 15964709

[ref49] Lageman S. K. Cerhan J. H. Locke D. E. C. Anderson S. K. Wu W. Brown P. D. (2010). Comparing neuropsychological tasks to optimize brief cognitive batteries for brain tumor clinical trials. J. Neuro-Oncol. 96, 271–276. doi: 10.1007/s11060-009-9960-y, PMID: 19618121

[ref50] Lezak M. (1995). Neuropsychological Assessment. 3rd. Oxford University Press: New York.

[ref51] Lonie J. A. Parra-Rodriguez M. A. Tierney K. M. Herrmann L. L. Donaghey C. O'Carroll R. E.. (2010). Predicting outcome in mild cognitive impairment: 4-year follow-up study. Br. J. Psychiatry 197, 135–140. doi: 10.1192/bjp.bp.110.077958, PMID: 20679266

[ref52] Merchant T. E. Conklin H. M. Wu S. Lustig R. H. Xiong X. (2009). Late effects of conformal radiation therapy for pediatric patients with low-grade glioma: prospective evaluation of cognitive, endocrine, and hearing deficits. J. Clin. Oncol. 27, 3691–3697. doi: 10.1200/JCO.2008.21.2738, PMID: 19581535 PMC2799064

[ref53] Meyers C. A. Boake C. (1993). Neurobehavioral disorders in brain tumor patients: rehabilitation strategies. Cancer Bull. 45, 62–64.

[ref54] Meyers C. A. Brown P. D. (2006). Role and relevance of neurocognitive assessment in clinical trials of patients with CNS tumors. J. Clin. Oncol. 24, 1305–1309. doi: 10.1200/JCO.2005.04.6086, PMID: 16525186

[ref55] Obayashi S. Matsushima E. Ando H. Ando K. Kojima T. (2003). Exploratory eye movements during the Benton visual retention test: characteristics of visual behavior in schizophrenia. Psychiatry Clin. Neurosci. 57, 409–415. doi: 10.1046/j.1440-1819.2003.01140.x, PMID: 12839523

[ref56] Okoukoni C. McTyre E. R. Ayala Peacock D. N. Peiffer A. M. Strowd R. Cramer C.. (2017). Hippocampal dose volume histogram predicts Hopkins verbal learning test scores after brain irradiation. Adv. Radiat. Oncol. 2, 624–629. doi: 10.1016/j.adro.2017.08.013, PMID: 29204530 PMC5707405

[ref57] Pendergrass J. Targum S. D. Harrison J. E. (2018). Cognitive impairment associated with cancer: a brief review, innovations clinical neuroscience. Innov. Clin. Neurosci. 15, 36–44. 29497579 PMC5819720

[ref58] Rao S. M. Bieliauskas L. A. (1983). Cognitive rehabilitation two and one-half years post right temporal lobectomy. J. Clin. Neuropsychol. 5, 313–320. doi: 10.1080/01688638308401179, PMID: 6643685

[ref59] Reyes-Botero G. Mokhtari K. Martin-Duverneuil N. Delattre J. Y. Laigle-Donadey F. (2012). Adult Brainstem Gliomas. Oncologist 17, 388–397. doi: 10.1634/theoncologist.2011-0335, PMID: 22382458 PMC3316925

[ref60] Ritchie K. Artero S. Touchon J. (2001). Classification criteria for mild cognitive impairment: a population-based validation study. Neurology 56, 37–42. doi: 10.1212/WNL.56.1.37 11148233

[ref61] Ruge M. I. Ilmberger J. Tonn J. C. Kreth F. W. (2011). Health-related quality of life and cognitive functioning in adult patients with supratentorial WHO grade II glioma: status prior to therapy. J. Neuro-Oncol. 103, 129–136. doi: 10.1007/s11060-010-0364-9, PMID: 20820874

[ref62] Santini B. Talacchi A. Squintani G. Casagrande F. Capasso R. Miceli G. (2012). Cognitive outcome after awake surgery for tumors in language areas. J. Neuro-Oncol. 108, 319–326. doi: 10.1007/s11060-012-0817-4, PMID: 22350433

[ref63] Schagen S. B. Klein M. Reijneveld J. C. Brain E. Deprez S. Joly F.. (2014). Monitoring and optimising cognitive function in cancer patients: present knowledge and future directions. Eur. J. Cancer Suppl. 12, 29–40. doi: 10.1016/j.ejcsup.2014.03.003, PMID: 26217164 PMC4250534

[ref64] Schenkenberg T. Bradford D. C. Ajax E. T. (1980). Line bisection and unilateral visual neglect in patients with neurologic impairment. Neurology 30, 509–517. doi: 10.1007/978-3-319-57111-9_194, PMID: 7189256

[ref65] Seigers R. Fardell J. E. (2011). Neurobiological basis of chemotherapy-induced cognitive impairment: a review of rodent research. Neurosci. Biobehav. Rev. 35, 729–741. doi: 10.1016/j.neubiorev.2010.09.006, PMID: 20869395

[ref66] Seo E. H. Lee D. Y. Choo I. H. Youn J. C. Kim K. W. Jhoo J. H.. (2007). Performance on the Benton visual retention test in an educationally diverse elderly population. J. Gerontol. B Psychol. Sci. Soc. Sci. 62, P191–P193. doi: 10.1093/geronb/62.3.p191 17507588

[ref67] Sheline G. E. Wara W. M. Smith V. (1980). Therapeutic irradiation and brain injury. Int. J. Radiat. Oncol. Biol. Phys. 6, 1215–1228. doi: 10.1016/0360-3016(80)90175-3 7007303

[ref68] Sherer M. Meyers C. A. Bergloff P. (1997). Efficacy of postacute brain injury rehabilitation for patients with primary malignant brain tumors. Cancer 80, 250–257. doi: 10.1002/(SICI)1097-0142(19970715)80:2<250::AID-CNCR13>3.0.CO;2-T, PMID: 9217038

[ref69] Sherman J. C. Colvin M. K. Mancuso S. M. Batchelor T. T. Oh K. S. Loeffler J. S.. (2016). Neurocognitive effects of proton radiation therapy in adults with low-grade glioma. J. Neuro-Oncol. 126, 157–164. doi: 10.1007/s11060-015-1952-5, PMID: 26498439 PMC12895352

[ref70] Stieber V. W. Mehta M. P. (2007). Advances in radiation therapy for Brain tumors. Neurol. Clin. 25, 1005–1033. doi: 10.1016/j.ncl.2007.07.005 17964024

[ref71] Stratta P. Mancini F. Mattel P. Daneluzzo E. Bustini M. Casacchia M.. (1997). Remediation of Wisconsin card sorting test performance in schizophrenia: a controlled study. Psychopathology 30, 59–66. doi: 10.1159/000285030 9168560

[ref1003] Stucki G. (2005). International Classification of Functioning, Disability, and Health (ICF). A Promising Framework and Classification for Rehabilitation Medicine. Am J Phys Med Rehabil 84, 733–740. doi: 10.1097/01phm.0000179521.70639.83 16205428

[ref72] Stuss D. T. Floden D. Alexander M. P. Levine B. Katz D. (2001). Stroop performance in focal lesion patients: dissociation of processes and frontal lobe lesion location. Neuropsychologia 39, 771–786. doi: 10.1016/S0028-3932(01)00013-6, PMID: 11369401

[ref73] Tallet A. V. Azria D. Barlesi F. Spano J. P. Carpentier A. F. Gonçalves A.. (2012). Neurocognitive function impairment after whole brain radiotherapy for brain metastases: actual assessment. Radiat. Oncol. 7:1. doi: 10.1186/1748-717X-7-77, PMID: 22640600 PMC3403847

[ref74] Taphoorn M. J. B. Claassens L. Aaronson N. K. Coens C. Mauer M. Osoba D.. (2010). An international validation study of the EORTC brain cancer module (EORTC QLQ-BN20) for assessing health-related quality of life and symptoms in brain cancer patients. Eur. J. Cancer 46, 1033–1040. doi: 10.1016/j.ejca.2010.01.012, PMID: 20181476

[ref75] Taphoorn M. J. B. Heimans J. J. Snoek F. J. Lindeboom J. Oosterink B. Wolbers J. G.. (1992). Assessment of quality of life in patients treated for low-grade glioma: a preliminary report. J. Neurol. Neurosurg. Psychiatry 55, 372–376. doi: 10.1136/jnnp.55.5.372, PMID: 1602310 PMC489078

[ref76] Taphoorn M. J. B. Klein M. (2004). Cognitive deficits in adult patients with brain tumours. Lancet Neurol. 3, 159–168. doi: 10.1016/S1474-4422(04)00680-5 14980531

[ref77] Tombaugh T. N. Mclntyre N. J. (1992). The mini-mental state examination. Progress Geriatr. 40, 922–935. doi: 10.1111/j.1532-5415.1992.tb01992.x 1512391

[ref78] Van Coevorden-van Loon E. M. P. Heijenbrok-Kal M. Loon W. Bent M. Vincent A. Koning I.. (2015). Assessment methods and prevalence of cognitive dysfunction in patients with low-grade glioma: a systematic review. J. Rehabil. Med. 47, 481–488. doi: 10.2340/16501977-1975, PMID: 25994416

[ref79] Vardy J. Wefel J. S. Ahles T. Tannock I. F. Schagen S. B. (2008). Cancer and cancer-therapy related cognitive dysfunction: an international perspective from the Venice cognitive workshop. Ann. Oncol. 19, 623–629. doi: 10.1093/annonc/mdm500, PMID: 17974553

[ref80] Vargo M. Henriksson R. Salander P. (2016). “Rehabilitation of patients with glioma” in Handbook of Clinical Neurology. 1st ed (Amsterdam: Elsevier B.V) 10.1016/B978-0-12-802997-8.00017-7 26948361

[ref81] Wefel J. S. Saleeba A. K. Buzdar A. U. Meyers C. A. (2010). Acute and late onset cognitive dysfunction associated with chemotherapy in women with breast cancer. Cancer 116, 3348–3356. doi: 10.1002/cncr.25098, PMID: 20564075

[ref82] Wefel J. S. Vardy J. Ahles T. Schagen S. B. (2011a). International cognition and cancer task force recommendations to harmonise studies of cognitive function in patients with cancer. Lancet Oncol. 12, 703–708. doi: 10.1016/S1470-2045(10)70294-1, PMID: 21354373

[ref83] Woods S. Delis D. Scott J. Kramer J. Holdnack J. (2006). The California verbal learning test–second edition: test-retest reliability, practice effects, and reliable change indices for the standard and alternate forms. Arch. Clin. Neuropsychol. 21, 413–420. doi: 10.1016/j.acn.2006.06.002, PMID: 16843636

[ref1004] World Health Organization. International Classification of Diseases (ICD-10) (2020). Available online: http://www.who.int/ classifications/icd/en/ (Accessed January 1, 2020).

[ref84] Yamamoto A. K. Sanjuán A. Pope R. Parker J. O. Hope T. M. H. Prejawa S.. (2022). The effect of right temporal lobe gliomas on left and right hemisphere neural processing during speech perception and production tasks. Front. Hum. Neurosci. 16: 803163. doi: 10.3389/fnhum.2022.803163, PMID: 35652007 PMC9148966

[ref85] Zhao Q. Guo Q. Li F. Zhou Y. Wang B. Hong Z. (2013). The Shape Trail test: application of a new variant of the trail making test. PLoS One 8:e57333. doi: 10.1371/journal.pone.0057333, PMID: 23437370 PMC3577727

[ref87] Zucchella C. Bartolo M. di Lorenzo C. Villani V. Pace A. (2013). Cognitive impairment in primary brain tumors outpatients: a prospective cross-sectional survey. J. Neuro-Oncol. 112, 455–460. doi: 10.1007/s11060-013-1076-8, PMID: 23417320

